# Structural and Functional Analysis of Female Sex Hormones against SARS-CoV-2 Cell Entry

**DOI:** 10.3390/ijms222111508

**Published:** 2021-10-26

**Authors:** Jorge Alberto Aguilar-Pineda, Mazen Albaghdadi, Wanlin Jiang, Karin J. Vera-Lopez, Rita Nieto-Montesinos, Karla Lucia F. Alvarez, Gonzalo Davila Del-Carpio, Badhin Gómez, Mark E. Lindsay, Rajeev Malhotra, Christian L. Lino Cardenas

**Affiliations:** 1 Laboratory of Genomics and Neurovascular Diseases, Vicerrectorado de Investigación, Universidad Católica de Santa María, Arequipa 04001, Peru; jaguilar@ucsm.edu.pe (J.A.A.-P.); kvera@ucsm.edu.pe (K.J.V.-L.); rnieto@ucsm.edu.pe (R.N.-M.); kalvarezf@ucsm.edu.pe (K.L.F.A.); gdavilad@ucsm.edu.pe (G.D.D.-C.); bgomez@ucsm.edu.pe (B.G.); 2 Cardiovascular Research Center, Cardiology Division, Massachusetts General Hospital, Harvard Medical School, Boston, MA 02114, USA; malbaghdadi@mgh.harvard.edu (M.A.); wjiang0@mgh.harvard.edu (W.J.); lindsay.mark@mgh.harvard.edu (M.E.L.)

**Keywords:** COVID-19, ACE2, sex hormones, estrogenes

## Abstract

Emerging evidence suggests that males are more susceptible to severe infection by the SARS-CoV-2 virus than females. A variety of mechanisms may underlie the observed gender-related disparities including differences in sex hormones. However, the precise mechanisms by which female sex hormones may provide protection against SARS-CoV-2 infectivity remains unknown. Here we report new insights into the molecular basis of the interactions between the SARS-CoV-2 spike (S) protein and the human ACE2 receptor. We further report that glycosylation of the ACE2 receptor enhances SARS-CoV-2 infectivity. Importantly, estrogens can disrupt glycan–glycan interactions and glycan–protein interactions between the human ACE2 and the SARS-CoV-2 thereby blocking its entry into cells. In a mouse model of COVID-19, estrogens reduced ACE2 glycosylation and thereby alveolar uptake of the SARS-CoV-2 spike protein. These results shed light on a putative mechanism whereby female sex hormones may provide protection from developing severe infection and could inform the development of future therapies against COVID-19.

## 1. Introduction

The novel coronavirus disease 2019 (COVID-19) global pandemic caused by infection with the severe acute respiratory syndrome coronavirus 2 (SARS-CoV-2) virus has infected nearly 200 million people worldwide resulting in nearly 5 million deaths as of 21 September 2021. Emerging data suggests that males are more susceptible to COVID-19 infection and are at higher risk of critical illness and death than females [1,2,3]. There has been consistent evidence of an increased case fatality rate (CFR) among males in nearly every country with available sex-disaggregated data including Peru, France, Greece, Italy, Mexico, Pakistan, Philippines, and Spain amounting to a 1.7 times higher CFR than females [4]. Indeed, sex differences in outcomes between men and women are known as evidenced by a meta-analysis that identified male sex as a risk factor for death and ITU admission [5]. Interestingly, testosterone-deprivation therapy for prostate cancer has been associated with improved outcomes for COVID-19, suggesting that suppression of the immune response by testosterone, as well as the protective effect of estrogen, may underlie the observed sex bias [6].

Understanding the mechanisms underlying enhanced COVID-19 susceptibility and disease severity in males is key to developing new therapies and guiding vaccine development. Changes in sex hormone concentration over an individual’s lifetime and associated risk of comorbid conditions, such as cardiovascular diseases, may also contribute to variability in disease susceptibility and severity [7]. It has been postulated that the male-biased sex divergence in COVID-19 deaths could be, in part, explained by the strict relationship between sex hormones and the expression of the entry receptor for SARS-CoV-2, the angiotensin converting enzyme 2 (ACE2) receptor [2,8]. Molecular studies have demonstrated that the male hormone testosterone regulates the expression of ACE2 and the transmembrane serine protease 2 (TMPRSS2) which is an androgen-responsive serine protease that cleaves the SARS-CoV-2 spike (S) protein and facilitates viral entry via ACE2 binding [9,10,11]. Androgen-driven upregulation of ACE2 levels may therefore be associated with increased vulnerability to severe infections in male patients with COVID-19. Paradoxically, ACE2 plays an important role in lung protection during injury which is attenuated by the binding of SARS-CoV-2 [12].

The presence of a male-biased dependence in COVID-19 susceptibility may imply the presence of a protective factor against SARS-CoV-2 infectivity in women. In addition to the ability of sex hormones to modulate the expression of proteins related to entry into host cells, both estrogens and androgens are also able to directly modulate immune cell function via receptor-mediated effects [13,14]. Additionally, sex chromosomes may mediate more favorable outcomes among women compared to men affected with COVID-19 [15]. X-linked genes associated with immune function tend to be expressed more often in females who generally have two X chromosomes compared to males [16]. 

Here, we examine the role of two estrogen molecules (17β-diol and S-equol) to modulate the ACE2-dependent membrane fusion protein and reduce cell entry of the SARS-CoV-2 spike protein into lung cells. To the best of our knowledge, we report new findings regarding the importance of molecular interactions between hACE2 and the viral spike (S) protein through site-specific glycosylation. Furthermore, we provide insights into the molecular basis for our observations that estrogens impair SARS-CoV-2 entry and highlight the potential for estrogens as an agent in patients with COVID-19.

## 2. Results

### 2.1. Glycosylation Site-Mapping of Human ACE2 and SARS-CoV-2 Spike Interactions

**Figure 1:**
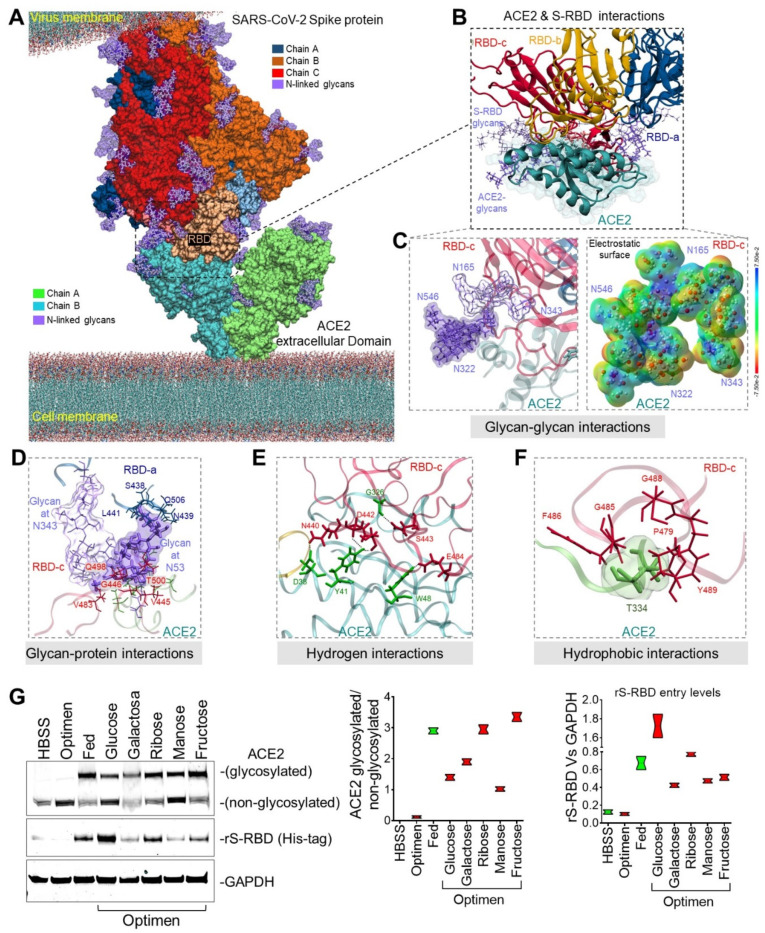
**Molecular bases of glycosylated hACE2 and SARS-CoV-2 Spike protein complex.** (**A**) 3D membrane surface representation of glycosylated ACE2 in complex with the SARS-CoV-2 Spike protein. (**B**) Close up of the interacting environment between ACE2 and the S-RBD trimer. (**C**) The left panel demonstrates glycan–glycan interactions between ACE2 (dark purple surface) and S-RBDc (light purple surface). The right panel shows that glycan–glycan contacts do not affect their molecular electrostatic potentials (MEPs) properties. The energy scale ranges from −0.075 μa (red) to 0.075 μa (blue). (**D**) ACE2 glycan at N53 forms glycan–protein contact with residues on the S-RBDa and S-RBDc proteins. (**E**) The ACE2 glycosylation induces the formation of hydrogen bonds that engages the helix α1 in the binding with multiple residues on the S-RBDc. (**F**) Hydrophobic interactions occur between ACE2 at T334 and multiple residues on the S-RBDc. (**G**) Immunoblot shows the glycosylation status of the human ACE2 in HUVECs treated with different saccharides. Glucose-treated cells induced the greatest internalization of the recombinant S-RBD. Quantification of protein levels of three replicate experiments is shown. Student’s *T*-test, 2 tails. Bar graphs are presented as mean with error bars (±SD).

Recent studies [17,18] have shown the ability of the SARS-CoV-2 virus to utilize a highly glycosylated spike (S) protein to elude the host’s immune system and bind to its target membrane receptor, ACE2, thus enabling entry into human cells. Based on the structural complementarity and steric impediments between the S protein and human ACE2 (hACE2) protein membranes, we mapped the glycosylation sites of both models [18,19,20,21] and performed molecular dynamics simulations (MDS) for 350 ns to allow the opening of the viral trimer and thus stabilize the glycosylated SARS-CoV-2 spike (S) (Figures S1 and S2, see Supplementary Materials) and hACE2 complex (Table S1, Figure S3 and Figure 1a). These analyses revealed that glycosylation of the ACE2 protein increases the affinity of the virus S protein to interact with the receptor via glycan–glycan interactions, glycan–protein interactions, hydrogen, and hydrophobic bonds (Table S2 and Figure 1b). Notably, glycan–glycan interactions occur between the ACE2 glycan at N322 and N546 and glycans found on the spike’s receptor binding domain (S-RBD) at N165 and N343 (Figure 1c, left panel). Despite the close interaction between ACE2 and S-RBD glycans, their affinity to anchor with highly negatively charged molecules such as the ACE2 protein remains unalterable (Figure 1c, right panel) suggesting that glycan and electrostatic-dependent surface tethering may represent a plausible mechanism for ACE-S-RBD binding and cell infection. The glycan–protein interactions occur between the ACE2 glycan at N53 and the residues of the S-RBD at N437, S438, N439, L441, V445, G446, V483, Q498, T500, and Q506 (Figure 1d). While ACE2 residues at D38, Y41, W48, and G326 form hydrogen bonds with residues of the S-RBD at N440, D442, S443, N450, and E484 (Figure 1f). Multiple distinct clusters of hydrophobic residues at the ACE2 surface were also found to interact with the S-RBD protein (Figure S4). Importantly, one key hydrophobic region on the ACE2 surface at T334 interacts with five residues of the S-RBD (P479, G485, F486, G488, and Y489) (Figure 1g). Given the insights afforded by our in silico MDS experiments, we sought to explore the impact of ACE2 glycosylation on S-RBD cell entry using cultured human umbilical vein endothelial cells (HUVECs). A variety of saccharide substrates were utilized for their ability to modulate glycosylation profiles in cells. The glycosylation pattern of the endogenous ACE2 was increased in nearly all treated cells (Figure 1h). Notably, co-incubation of HUVECs with 10 ug of recombinant S-RBD (rS-RBD) protein revealed that glucose (25 mM) pretreatment was associated with the greatest degree of rS-RBD entry into the cells by ~eight-fold compared with unfed (Optimen) cells (Figure 1g). This model indicates that glycosylated residues surrounding the cavity at the top of the ACE2 molecule could increase binding by the S-RBD. Given the possibility that occupancy at glycosylated residues or S-RBD binding sites by estrogens could modify the affinity of the SARS-CoV-2 virus and alter entry into the cell thereby reducing infectivity, we sought to further examine these interactions using a range of complementary experimental approaches. 

### 2.2. Estrogens Bind to ACE2 and Stimulate Its Stabilization and Internalization

**Figure 2:**
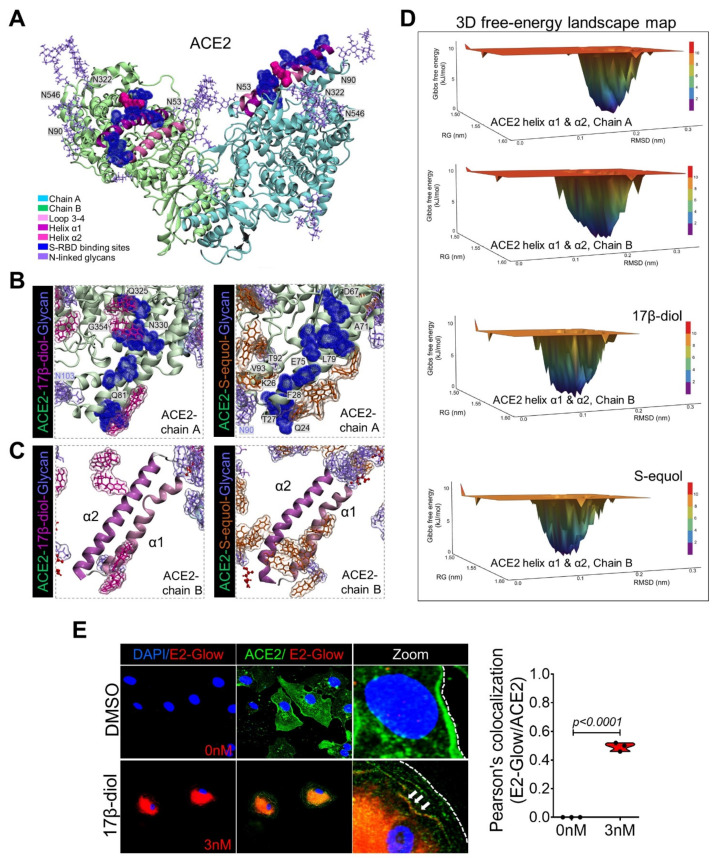
**Estrogen effects on ACE2 structural energy.** (**A**) 3D representation of the human ACE2 glycosylated residues and key regions used by the SARS-CoV-2 S protein to mediate entry into cells. S-RBD-binding sites are colored in dark blue and glycans in purple. (**B**) 3D molecular interactions between ACE2 and 17β-diol (magenta) or S-equol (orange) molecules obtained by 100 ns of molecular dynamics simulations (MDS). (**C**) A plain representation of solvated ACE2-helix α1 and α2 substructures by estrogen molecules. (**D**) FEL maps represent the conformational energy of helix α1 and α2 substructures with estrogen molecules during MDS (last 20 ns of MDS). The energy scale ranges from 12 kJ/mol (red) to 0 kJ/mol (blue). (**E**) Immunofluorescence microscopy analysis on HUVECs cells treated with or without conjugated 17β-diol (E2-Glow), shows colocalization (yellow) between ACE2 (green) and 17β-diol (red).

**Figure 3:**
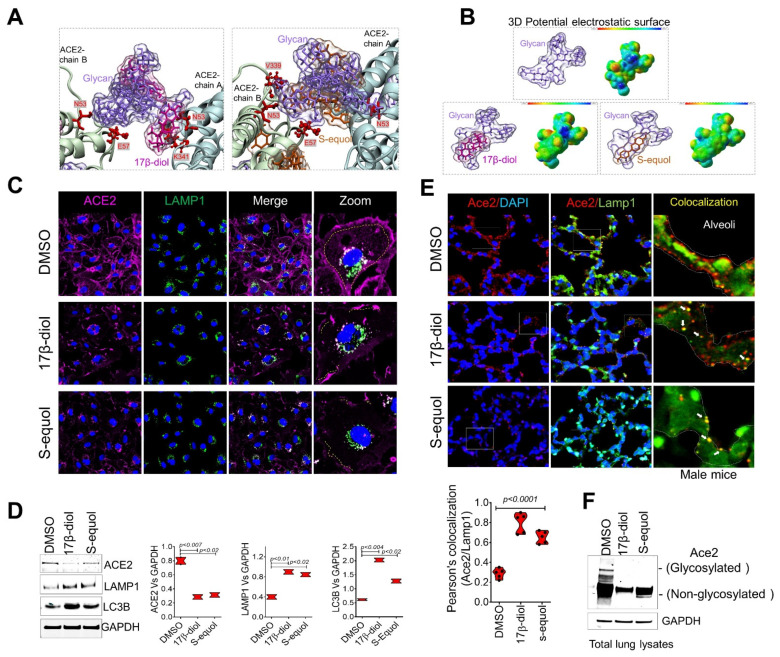
**Estrogens bind to ACE2 glycans to promote its internalization.** (**A**) Glycan–estrogen interactions stabilize ACE2 Glycan-residues at E57, N53, K341, and V339 (red color). (**B**) MEP maps show the electrostatic impact of estrogen molecules on the surface of ACE2 glycans. The energy scale ranges from −0.075 μa (red) to 0.075 μa (blue). (**C**) Immunofluorescence staining of human ACE2 (magenta) and the lysosome marker LAMP1 (green), shows loss of ACE2 membrane levels in HUVECs treated with 17β-diol or S-equol compared with the control group (DMSO). (**D**) Immunoblot shows decreased levels of total ACE2 protein which associates with increased endocytosis activity as evidenced by immunoblot for LC3b and LAMP1. (**E**) Histologic analysis of wild-type mouse lungs after 48 h of intratracheal instillation with 17β-diol or S-equol shows loss of Ace2 signal (red) on the membrane of alveoli cells. Estrogen-treated lungs show greater Ace2-Lamp1 colocalization (white arrows) indicating internalization of the receptor. (**F**) Immunoblot shows decreased levels of total and glycosylated Ace2 proteins in estrogen-treated lungs from male mice. Quantification of protein levels of three replicate experiments is shown. Student’s *t*-test, 2 tails. Bar graphs are presented as mean with error bars (±SD).

In an effort to explore the potential protective effects of female sex hormones against SARS-CoV-2 infection, we examined the impact of estradiol (17β-diol) and a dietary-derived phytoestrogen (S-equol) on hACE2 structure and protein expression by a combination of in silico modeling, in vitro, and in vivo analysis. Specifically, in light of the importance of glycan–glycan interactions that mediate virus-ACE2 interactions, we sought to analyze the effect of estrogens on key molecular viral and receptor binding sites. In agreement with a previous report [19], we identified three important regions on the ACE2 surface that are utilized for SARS-CoV-2 binding. The environment of these regions is composed of a high density of glycans, including a helix α1 from residues I21 to T52, a helix α2 from residues V59 to M82, and one loop from residues K353 to G354 (Figure S4 and Figure 2a). We then homogeneously solvated the glycosylated hACE2 structure with 60 molecules of 17β-diol or S-equol followed by 150 ns of MDS (Figure S5a,c,d). Remarkably we found that the 17β-diol molecules interact with residues at F28, Y41, Q76, T78, Q81, M82, and the S-equol molecules interact with residues at Q24, K26, T27, F28, K31, E35, L39, N64, D67, K68, A71, F72, E75, Q76, and L79 (Figure 2b,c, Table S4). Both estrogen molecules energetically stabilized the α1 and α2 helices by physical interactions and thereby minimized the fluctuation of the ACE2 chains A and B (Figure 2c and Figure S5b,d). Importantly, our calculation of free-energy landscape (FEL), demonstrated that the surface of chain B of ACE2 (S-RBD’s preferred interaction region) loses its interaction energy with the S-RBD protein from 10.2 kJ/mol to 8.58 kJ/mol (16%) for the 17β-diol system and to 9.18 kJ/mol (10%) for the S-equol system (Figure 2d). To support our in silico observations, we treated HUVECs with 3 nM of 17β-diol labeled (E2-Glow) with a low molecular weight fluorophore for 6 h under physiological conditions. Microscopy analysis demonstrated the colocalization between the E2-Glow and the ACE2 receptor (Figure 2e). In addition, binding of either estrogen molecules to the surrounding hydrophobic pocket of ACE2 at the residue T334 promotes a decrease in energy by ~12% which may have a negative impact on the attachment of the S-RBD protein to the receptor (Figure S6). We also observed estrogen–glycan interactions particularly at the glycan–protein interactions between the ACE2 (N53) and the S-RBD (N432) (Figure 3a). Indeed, glycans are highly polar structures due to their high content of hydroxyl groups which make them suitable for attachment to the ACE2 protein (mostly negatively charged) or the SARS-CoV-2 S protein (polarly charged). The density functional theory (DFT) calculation shows an important decrease of the glycan’s molecular electrostatic potential (MEP) due to the interactions with either estrogen molecules. Therefore, estrogen–glycan interactions could decrease the adhesive effect of glycans that enhance S-RBD and ACE2 receptor interactions (Figure S7 and Figure 3b). These structural analyses suggest that estrogens could act as putative ACE2 ligands due to their ability to bind to highly energetic pockets at the top of the ACE2 surface protein which may increase its conformational equilibria and potentially boost its internalization to the cytoplasm. To support our in silico analyses, we treated HUVECs with 17β-diol (3 nM) or S-equol (10 nM) overnight under normal physiologic conditions. Immunofluorescent staining demonstrated that estrogen-treated cells have less ACE2 membrane cellular localization (Figure 3c). Immunoblot analysis revealed that endogenous and dietary estrogens promote ACE2 internalization and degradation through the endocytosis process as assessed by LC3b [22] and LAMP1 [23] protein activation in treated cells (Figure 3d). To test the hypothesis that lower levels of estrogens are associated with increased levels of ACE2 protein in the respiratory tract, we administrated intratracheally either 17β-diol (0.3 μM) or S-equol (1 μM) to male mice. Histologic analysis of lung sections confirmed that both forms of estrogens decrease ACE2 membrane expression levels in lung alveoli and also reduced the glycosylation of the ACE2 receptor (Figure 3e,f).

### 2.3. Estrogens Interfere with SARS-CoV-2 Receptor Binding and Block Entry into the Cell

**Figure 4:**
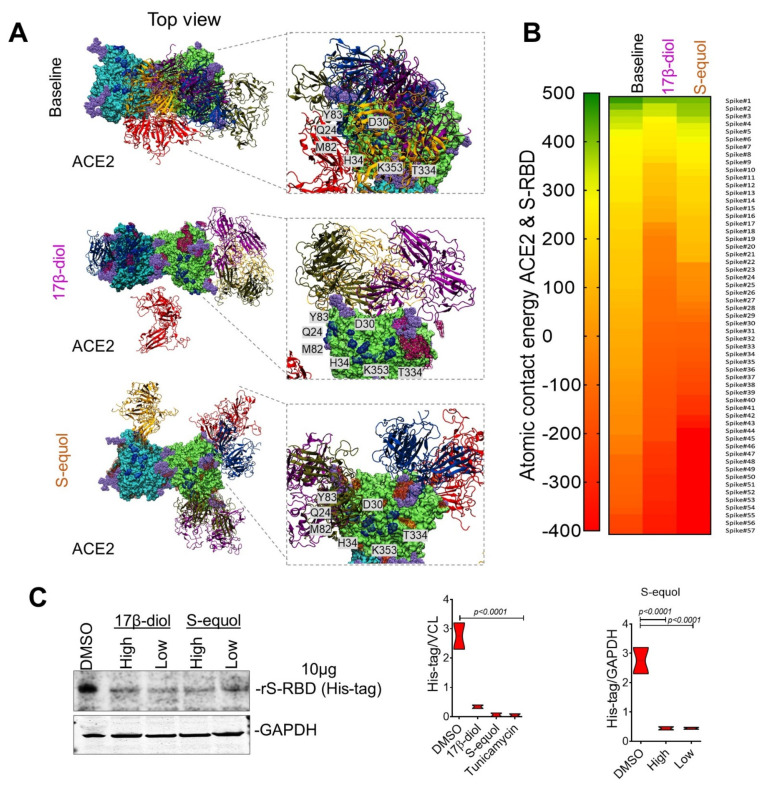
**Estrogen’s impact on ACE2 and S-RBD interactions.** (**A**) Top view of the 3D ACE2 surface interacting with 5 top-scored S-RBDs (top 1—blue, 2—red, 3—orange, 4—purple, and top 5—yellow). S-RBDs were scored based on shape complementarity principles. (**B**) Heatmap of atomic contact energy between ACE2 and 57 S-RBDs shows spontaneous energy structures from most favorable (green) to less favorable S-RBD structures (red). Energy scale ranging from 500 Kcal/mol to −500 Kcal/mol. (**C**) Immunoblot of isolated proteins from cultured HUVECs shows a 90% inhibition of S-RBD entry into cells in estrogen-treated cells. Quantification of protein levels of three replicate experiments is shown. Student’s *T*-test, 2 tails. Bar graphs are presented as mean with error bars (±SD).

To determine if the decline of conformational Gibbs free energy and gain in stabilization of ACE2 due to estrogen binding could affect the ability of the S protein to interact with the ACE2 receptor and thereby its entry into cells, we performed a refinement step of ACE2-free or ACE2-estrogen models with 100 ns of MDS followed by molecular docking with the SARS-CoV-2 S protein. From 241 structures obtained, 57 with top scores were chosen for further analysis (Tables S5 and S6). The ACE2-17β-diol model promoted the shift of S-RBDs from the binding surface toward the lateral side of the ACE2 protein decreasing the number of contact residues. Notably, S-RBDs completely lose contact with key ACE2-glycosylated residues at N53, N103, N432, and N690. We also observed that the contact between the S-RBD and the helix α1 and α2 of ACE2 moved toward the N-terminal of the helix and thus affected the ability to bind the receptor. In the same manner, the ACE2-S-equol model demonstrated that S-equol blocks the contact between the S-RBDs and the receptor’s surface, notably promoting novel interactions at the C-terminal of the helix α2 causing nonspecific contacts with the receptor at residues Q429-I436 and P590-N601. Interestingly, we found that the 17β-diol interacts with 66 residues on the surface of the receptor and notably forms a cluster on glycans at N546 (chain A) and N322 (chain B). On the other hand, the S-equol molecules tend to interact more widely, accounting for a total of 145 interactions, including on 63 residues on chain A and 82 residues on chain B. (For better visualization, only the five top-scored S-RBD structures are shown in Figure 4a.) The nonspecific binding by the S-RBDs could be explained by the susceptibility of ACE2 to interact with polar molecules and especially to electrophilic attacks. The fact that the 17β-diol or S-equol contain few polar groups but are deficient in negative charge renders them more susceptible to attack the surface of hACE2 thereby blocking S-RBD from binding correctly (Figure S9). In addition, we computed the binding score of these models using the atomic energy contact function and in agreement with our previous docking results observed that both estrogen molecules significantly reduced the atomic energy contact between virus and receptor. Remarkably, the 17β-diol reduced the atomic contact by 80% and the S-equol by 65% indicating that the entry of the virus may be affected by the presence of either estrogen molecules (Figure 4b). To validate our in silico prediction, we pretreated HUVECs with either estrogen molecules followed by incubation with 10μg of rS-RBD protein overnight. Importantly, either low or high concentration of 17β-diol (low = 1 nM and high = 5 nM) or S-equol (low = 5 nM and high = 20 nM) blocked more than 90% of the rS-RBD protein entry into the cell cytoplasm of HUVECs as assessed by immunoblot, for both estrogen-based treatments (Figure 4c). Together these results suggest a potential molecular mechanism by which estrogens may provide protection against severe infection in COVID19 among women and individuals with phytoestrogen intake.

### 2.4. Estrogens Block SARS-CoV-2 Infection of the Respiratory Tract in an Animal Model of COVID-19

**Figure 5:**
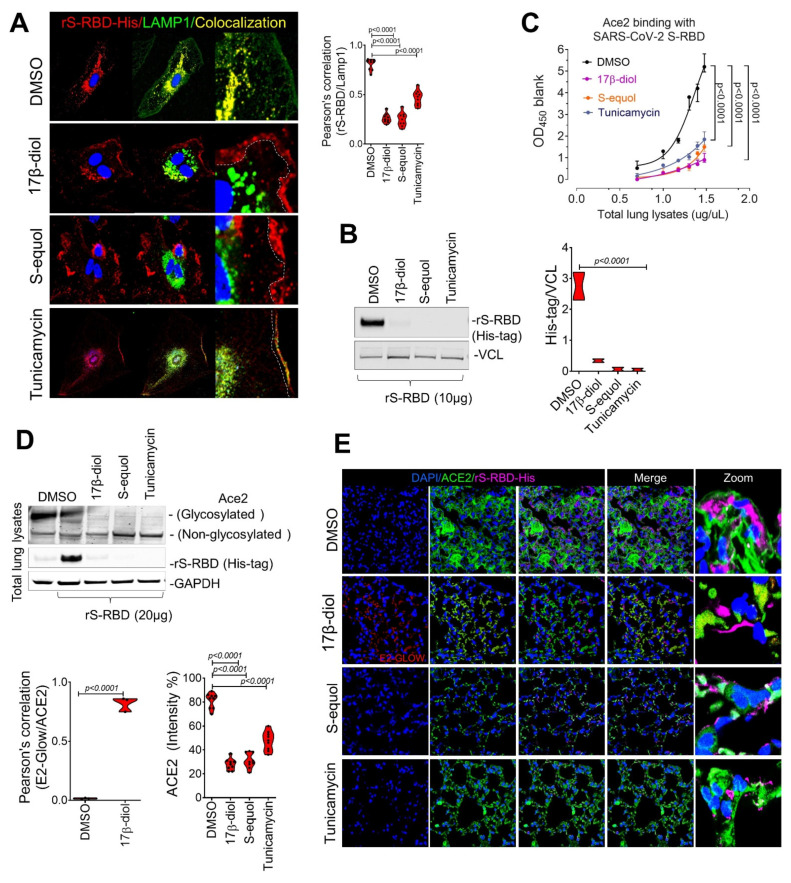
**Estrogens block SARS-CoV-2 S protein uptake in the respiratory tract in vivo.** (**A**,**B**) Immunofluorescence analysis of S-RBD entry into HUVECs pretreated estrogens (17β-diol or S-equol) or Tunicamycin (glycosylation inhibitor) followed by treatment with 10μg/mL of recombinant S-RBD (red) demonstrate that all 3 treatments cells reduced entry of S-RBD entry into cells via a reduction in Ace2 internalization as shown by colocalization with LAMP1 (green) and immunoblot. (**C**) ELISA-based binding assay using lung protein lysates from male mice (K18-ACE2) treated with 17β-diol (0.3 μM) or S-equol (1μM) or Tunicamycin (1 mg/Kg) shows reduced SARS-CoV-2 S protein affinity for the Ace2 receptor. (**D**) Immunoblot shows the glycosylation levels of Ace2 from total lung lysate in mice treated with estrogens or Tunicamycin. (**E**) Immunofluorescence of Ace2 (green) and RBD (magenta) in lungs from lysates from male mice (K18-ACE2) treated with estrogens or Tunicamycin.

Our findings have demonstrated that glycosylation of the ACE2 receptor is a critical step in the interaction between the virus and host cells. To determine if the disruption of ACE2 glycosylation could affect SARS-CoV-2 entry into cells, we treated HUVECs with both estrogen molecules (17β-diol or S-equol) or Tunicamycin (0.2μM), an inhibitor of glycosylation, for 24 h under physiological conditions. Importantly, all three drugs blocked more than 90% of the rS-RBD protein entry into the cell cytoplasm as assessed by immunofluorescence and colocalization with LAMP1, a lysosome marker, and by immunoblot (Figure 5a,b). Next, we sought to test the ability of estrogens and Tunicamycin to block key interactions between ACE2 and the SARS-CoV-2 S protein and thereby infection of the respiratory tract using the animal model of COVID-19. Previous in vivo studies have demonstrated that ovariectomized female mice increased the expression of ACE2 receptor thereby favoring the viral entry [24,25,26]. Whereas castration, which reduces androgen levels, resulted in the downregulation of ACE2 [6]. Since androgen administration was closely involved in the transcriptional activation of ACE2, we used male mice overexpressing the human ACE2 to enhance the interactions of the rS-RBD protein and the ACE2 receptor. Thus male mice were treated with 17β-diol (0.3 μM) or S-equol (1 μM) via intratracheal instillation or Tunicamycin (1 mg/kg) via intraperitoneal injection for 24 h before tissue collection. An ELISA-based binding assay showed a significant decrease of ACE2 affinity to SARS-CoV-2 S protein in lungs from mice treated with either estrogen molecules or glycosylation inhibitor compared with the control group (Figure 5c). We then evaluated in vivo whether intratracheal administration of estrogen or tunicamycin would reduce the internalization of the S protein in lung tissue from male mice. We observed that pretreatment with estrogen molecules or Tunicamycin 24 h before intratracheal instillation of rS-RBD (20μg, overnight treatment) decreased the intake of the rS-RBD by lung alveolar cells which was associated with lower levels of glycosylated ACE2 (Figure 5d). Immunofluorescence microscopy showed an increased signal for the rS-RBD on the surface of lung cells which likely results from reduced binding to ACE2 in treated mice compared to the untreated group. In contrast, control (DMSO-treated) lungs showed normal ACE2 membrane localization and cytoplasmic r-S-RBD signal indicating the unperturbed uptake of the rS-RBD protein (Figure 5e). The observed increase in extracellular rS-RBD in alveolar cells from lungs of treated mice suggests that either glycosylation disruption or estrogen treatment reduces internalization of the S protein. Indeed, we observed that pretreatment resulted in rS-RBD protein accumulation on the surface of the alveolar cells (Figure 5d) rather than being internalized into the cytoplasm which would thereby support viral replication and disease progression. Our data demonstrate that estrogens or disruption of glycosylation may interfere with SARS-Cov-2 infection in the respiratory tract through direct interaction with the Ace2 receptor in vivo. 

## 3. Discussion

Increased susceptibility and risk of adverse clinical outcomes among males affected by COVID-19 has been reported in multiple epidemiological studies [4,7,8,9]. Sex-related hormones can effectively upregulate viral target proteins that may increase viral entry and pathogenicity in patients following exposure to the SARS-CoV-2 virus. A detailed understanding of the molecular and cellular mechanisms modulated by estrogen that contribute to viral pathogenicity is therefore critical to the development of new therapies to combat the COVID-19 pandemic. Besides the epidemiologic evidence suggesting that females are protected from severe infection, a recent study has demonstrated that the female reproductive tract expresses very low levels of the ACE2 receptor and almost undetectable TMPRSS2, suggesting that the virus is unlikely to infect the female reproductive tract, where female sex hormones are produced [27,28]. In the current study, we utilized in silico, in vitro, and in vivo studies to characterize important glycosylation-mediated interactions between the SARS-CoV-2 virus spike (S) protein and the human ACE2 receptor that can be modulated by endogenous or dietary estrogens in a manner that may be protective against the SARS-CoV-2 entry into human cells. 

Previous studies have highlighted the critical role of viral glycosylation in viral pathobiology, host immune system evasion, and infectivity in a range of human viral illnesses [29]. In many of these viruses, the viral envelope and secreted proteins are extensively glycosylated which is necessary for the structural integrity and functionality of these proteins. Viral proteins may be glycosylated by the host cell as viruses are able to hijack cellular glycosylation processes. However, little data exists on the impact of glycosylation of host proteins necessary for viral entry, such as ACE2, on viral infectivity. Using a novel molecular simulation approach, we demonstrated that ACE2 glycosylation augments binding of the viral S protein by supporting multiple types of interactions including glycan–glycan and glycan–protein interactions, thereby facilitating the stability and affinity of viral binding to the target host receptor. We extend these in silico observations by also demonstrating that entry of the rS-RBD can be augmented in vitro by exposure of cultured HUVECs to a hyperglycemic environment that increases ACE2 glycosylation. These observations provide insights into the enhanced susceptibility of diabetic patients to severe infections and death from COVID-19 [30,31,32]. Based on these findings that ACE2 glycosylation enhances interaction with the viral S protein in silico, we explored whether the predominant endogenous form of estrogen, 17β-diol, may provide a protective effect as assessed using molecular modeling in vitro and in vivo models of viral infectivity. In addition, we used an identical approach to understand the potential protective mechanisms of dietary phytoestrogens on SARS-CoV-2 infectivity observed in populations with low CFRs where consumption of these foods is high. We found that estrogens compete with the S-RBD protein to bind specific sites that are used by the virus to bind the receptor. Indeed, estrogens were found to bind at almost all sites including hACE2 glycans causing a reduction of energy on the surface of the receptor rendering the receptor less susceptible to interact with other molecules including the virus. Our findings that both endogenous and dietary estrogens interfere with S protein and ACE2 interactions in silico that is associated with reduced S protein uptake in a model of SARS-CoV-2 infectivity are consistent with prior studies demonstrating that estrogens have antiviral properties against HIV, Ebola, and hepatitis viruses [33]. Additional evidence showed that decreased levels of estrogens in postmenopausal women is an independent risk factor for severity in female COVID-19 patients [34]. The findings of the current study thus represent novel findings in our understanding of the molecular mechanisms underlying reduced susceptibility to SARS-CoV-2 among females and in countries where dietary estrogens are high. 

We demonstrate that ACE2 glycosylation augments the binding of the viral S protein by supporting multiple types of interactions including glycan–glycan and glycan–protein interactions, thereby facilitating the stability and affinity of viral binding to the target host receptor. These observations provide insights into the enhanced susceptibility of diabetic patients to severe infections and death in COVID-19 [32,33]. Based on our observations on the importance of glycosylation and identification of key hACE2 amino acids sites used by the SARS-CoV-2 virus and enable cell entry, we examined whether modulation of these key molecular interactions by estrogens may block SARS-CoV-2 infectivity observed in humans. We found that estrogens compete with the s-RBD protein to bind specific sites that are used by the virus to bind the receptor. Indeed, estrogens were found to bind at almost all sites including hACE2 glycans causing a reduction of energy on the surface of the receptor rendering the receptor less susceptible to interact with other molecules including the virus.

We then examined the ability of estrogen molecules to interfere with S protein uptake into pulmonary epithelial cells using an in vivo model of SARS-CoV-2 infectivity. In agreement with our cellular experiments, lung cells from mice treated with dietary or endogenous estrogens were not able to get infected by the rS-RBD. In addition, we observed a remarkable reduction of ACE2 binding possibly due to the low levels of hACE2 expression on the pulmonary epithelium in those mice treated with estrogen molecules. Our observation that estrogens reduce ACE2 glycosylation and are associated with reduced SARS-CoV-2 infectivity is consistent with previous investigations demonstrating the importance of SARS-CoV-2 spike protein glycosylation in viral pathogenicity [18]. Indeed, patients with diabetes in whom circulating levels of glucose and advanced glycosylation end products are elevated experience worse outcomes in COVID-19 [35]. We then demonstrated that pretreatment with the anti-glycosylation agent, Tunicamycin, was also associated with reduced SARS-CoV-2 infectivity highlighting the therapeutic potential for intensive glucose control in patients with COVID-19 as has been recently reported in patients with diabetes [36].

In conclusion, we provide a molecular basis that helps to elucidate the potential protective effect of estrogens in women infected by the SARS-CoV-2 virus which could inform the development of future therapeutic measures to protect against SARS-CoV-2 infection including the design of suitable blocking antibodies, estrogen-related treatments, and vaccine development. 

## 4. Methods

### 4.1. Immunofluorescence Microscopy

For immunofluorescence, HUVEC cells were cultured into 8-well Lab-TekTM II Chamber Slides (NuncTM, Thermofisher, CA, USA) and were then treated with either 17β-diol at 3 nM or S-equol at 10 nM. Cells were rinsed twice with ice-cold PBS, fixed with 4% paraformaldehyde in PBS (PFA, Boston, MA, Bioproducts) for 10 min at rt, and were permeabilized with 0.1% Triton-X (Sigma–Aldrich) for 3 min. The slides were blocked with 10% Donkey-serum, and 0.3 M glycine in PBS-Tween 20 (0.1%) for 1 h at rt. Subsequently, the antibodies anti-ACE2 (1:100), S-RBD-His-tag (1:50), anti-LAMP1 (1:50), and anti-LC3b (1:50) were added and slides were incubated overnight at 4 °C. The slides were then washed 3 times for 5 min each with PBS-T and were incubated with secondary antibodies at 1:400 dilution for 1 h at room temperature. Following immunostaining, slides were mounted with diamond mounting medium containing DAPI (Thermo Fisher, CA, USA). Slides were then visualized with the Leica TCS SP8 confocal microscopy station and the pictures were digitized with the Leica Application Suite X software. 

### 4.2. Protein Extraction and Immunoblot

HUVEC cells were rinsed twice with ice-cold PBS and proteins were extracted with M-PER for whole-cell lysis, respectively (Thermo Fisher, CA, USA). These lysis buffers contained Halt protease, phosphatase inhibitors, and EDTA (Thermo Fisher). The protein concentration was determined by the colorimetric bicinchoninic acid assay (BCA assay, Thermo Fisher, CA, USA). Equal amounts of total protein from cell lysates were separated by SDS-PAGE (25 μg or 40 μg for ACE2, LAMP1, LC3b, and rS-RBD-His-tag, respectively). Proteins from the gel were then electro-transferred onto 0.45 μm nitrocellulose and 0.2 μm PVDF membranes. The membranes were then blocked for 1 h at room temperature, with either 5% non-fat powdered milk dissolved in TBS-T or 5% bovine serum albumin in TBS-T, for the nitrocellulose and PVDF membranes, respectively. Following blocking, membranes were incubated overnight at 4 °C with the primary antibodies anti-ACE2 (1:1000), anti-LAMP1 (1:2000), anti-LC3b (1:1000), and anti-His-tag (1:1000). The Odyssey infrared western system was used to detect target proteins. Band intensity was quantified using ImageJ software.

### 4.3. Animal Treatment

All experiments involving mice were approved by the Partners Subcommittee on Research Animal Care. Personnel from the laboratory carried out all experimental protocols under strict guidelines to ensure careful and consistent handling of the mice.

*Mouse model of SARS-CoV-2 S protein entry*. Nine-week-old male C57BL/6 were purchased from The Jackson Laboratories, USA (Stock No: 034860). To administrate the recombinant S-RBD protein. Briefly, mice were anesthetized with sevoflurane inhalation (Abbott) and placed in dorsal recumbency. Transtracheal insertion of a 24-G animal feeding needle was used to instill estrogen molecules, rS-RBD, or vehicle (DMSO), in a volume of 80 µL. Mice were sacrificed 24 h after instillation of rS-RBS and lungs were removed for further analysis. 

*Histology.* Lungs were then fixed in formalin (10%) for 24 h before transfer to 70% ethanol for photography prior to paraffinization and sectioning (7 μM) and paraffin embedding. Slides were produced for tissue staining for quantitative analysis. 

### 4.4. In Vitro Treatment

*Saccharides treatment:* Hypoglycemic media was composed of HBSS buffer or Optiment media. Normal media contained complete endothelial cell growth media. For hyperglycemic media, Optiment was supplemented with D-glucose at 25 mM, D-galactose at 50 mM, D-ribose at 250 μM, D-mannose at 300 μM, or D-fructose at 20 μM. HUVECs at 60–70% confluence were supplemented with hypoglycemic, normal, or hyperglycemic media 24 h before incubation with 10 μg of recombinant S-RBD-His-tag overnight.

*Estrogen treatment*: HUVECs at 60–70% confluence were supplemented with opti-MEM 24 h before treatment with complete growing media containing 17β-diol (or E2-Glow from Jena Bioscience cat# PR-958S) at a concentration of 3 nM or S-equol at a concentration of 10 nM or Tunicamycin at 0.05 μM for 24 h. Fresh media containing rS-RBD (10 μg) was supplied the next day. Prior to cellular collection, cells were washed with sterile PBS, protein extraction was performed as described above.

### 4.5. rS-RBD-ACE2 Binding Assay

A total of 500 μg of total protein extracts from mouse lungs were cleaned up with IgA/IgG agarose beads for 1 h at 4 °C on a rotator followed by resuspension in assay diluent at 1×. Then 100 μL of each lysate containing 0, 5, 10, 15, 20, 25, or 30 μg of total protein were placed into the corresponding well of a COVID-19 S protein microplate (Cat#: CoV-SACE2, Ray Biotech, Inc. GA, USA) for overnight incubation at 4 °C on a rotator. Then the supernatant was removed, and wells were washed x 5 followed by incubation with 1× HRP-conjugated secondary antibody solution for 1 h at room temperature. Then 100 μL of TMB one-step substrate reagent was added to each well for 30 min at room temperature. Before read 50 μL of stop solution was added and the microplate was read at 450 nm.

### 4.6. Statistics

Results are given as mean ± SD. Student’s *t*-test (2-tailed) was applied to determine the statistical significance of difference between control and treated groups (* *p* < 0.05, ** *p* < 0.01, and *** *p* < 0.001). For all experiments, at least 3 experimental replicates were performed. Violin plot graphs show mean ± SD. Data were analyzed, and graphs were prepared with Prism 6.0 (GraphPad Software, San Diego, CA, USA). *p* values of less than 0.05 were considered statistically significant.

### 4.7. Model Building and Glycosylation Process

The crystalline structures used in this work were PDB ID:6VXX [20] for the SARS-CoV-2 spike protein (trimeric structure) and PDB ID:6M17 [19] for the ACE2 protein (dimeric structure), both obtained from the RCSB Protein Data Bank. Given that the 6VXX template structure was initially in its closed conformation, we first complete the missing residues using the Modeller software version 9.24, 8V2 [37] followed by the refining of the residues atomic positions using the Swiss-Model server (see Table S1 and Figure S1a). The glycosylation process was carried out using the glycan GlcNAc_2_Man_3_ template, a common core sugar-glycoside sequence composed of 2 N-acetyl glucosamines and 3 mannoses [38,39] (Figure S3b). The glycosylated spike protein was built using the OPLSAA based DoGlycans software [40]. Only 22 N-glycosylated residues were considered but the O-glycosylation sites were not included in this study (Figure S1b). Then 350 ns of molecular dynamic simulations were applied to adopt the open “up” conformation of the glycosylated spike protein (Movie S1 and Figure S1c). To build the extramembrane ACE2 dimeric protein (I21 to G732), we used the closed conformation of the receptor previously reporter [19]. The ACE2 structure contains two zinc ions in the peptidase domain which were considered in this work. The remaining missing residues of the ACE2 were added by using the Swiss-Model server [41]. For the glycosylated ACE2 receptor structure, we used the methodology described above and all N-glycosylated residues were considered followed by 250 ns of molecular dynamic simulations to stabilize the atomic conformation of the ACE2 protein (Figure S3c). 

### 4.8. Estrogens Solvated System 

To build the solvated estrogen-ACE2 systems, we first averaged the glycosylated ACE2 protein structures of the last 50 ns of a total of 250 ns of molecular dynamic simulation trajectories. Then we quantum optimized the 17β-diol and S-equol structures and the force fields were obtained using LigParGen server [42,43,44]. Before ACE2 solvation with the estrogen molecules, the volume of the simulation box was augmented by 0.4 nm in all directions. Once the protein was centered in the simulation box, 60 molecules of 17β-diol or S-equol (26.6 and 26.5 mM solutions, respectively) were added followed by water solvation using the gmx solvate module and TIP4P explicit water model [45] from GROMACS software version 2019.4 [46]. In the solvation process, we made sure that the estrogen molecules were not close to the protein at the start of the MD simulations.

### 4.9. Simulation Details

All quantum simulations were performed using density functional theory (DFT) [47] at B3LYP/TZVP level [48,49]. To understand the impact of the solvent (H_2_O) on the estrogen molecules, we used self-consistent reaction field (SCRF) theory. Additionally, the calculations were performed using the electronic structure program Gaussian 16 [50] and results were visualized by GaussView v. 6 [51]. Frequency analysis was used to optimize the 17β-diol and S-equol to ensure the global minimum potential energy conformation of estrogen molecules. These optimized structures were used in the molecular dynamic simulations. For MEP analysis, single-point calculations were carried out and total electron densities were mapped on molecular electrostatic potential surface. For all molecular interactions, molecular dynamics simulations were performed with OPLS/AA force field parameters [52] using GROMACS (v.2019.4) [46]. Na^+^ was used for the neutralization of total charge in the simulation box and NaCl at 150 mM was used to mimic physiological conditions. All molecular systems were built in a triclinic simulation box considering periodic boundary conditions (PBC) in all directions (*x*, *y*, and z). The distance of the protein’s surface to the edge of the periodic box was 1.5 nm for the ACE2 receptor and SARS-CoV-2 spike protein, and 2.3 nm for the ACE2-estrogen systems. A 1 fs step was applied to calculate the motion equations using the Leap-Frog integrator [53]. The temperature for proteins and water ions in all simulations was set at 309.65 K using the modified Berendsen thermostat (V-rescale algorithm) [54] with a coupling constant of $\tau_{T}=0.1$ ps. The pressure was maintained at 1 bar using the Parrinello–Rahman barostat [55] with a compressibility of 4.5 × 10^−5^ bar^−1^ and a coupling constant of $\tau_{P}=2.0$ ps. All simulations were carried out with a short-range non-bonded cut-off of 1.1 nm and the particle mesh Ewald (PME) method [56] was used to compute the long-range electrostatic interactions with a tolerance of 1 × 10^5^ for contribution in real space. The Verlet neighbor searching cut-off scheme was applied with a neighbor-list update frequency of 10 steps (20 fs). Bonds involving hydrogen atoms are constrained using the linear constraint solver (LINCS) algorithm [57]. Energy minimization in all simulations was performed with the *steepest descent* algorithm for a maximum of 100,000 steps. For the equilibration process, we performed two steps, first a 1 ns of dynamics in the NVT (isothermal-isochoric) ensemble followed by 2 ns in the NPT (isothermal-isobaric) ensemble. The final simulation was performed in the NPT ensemble for 350 ns for the SARS-CoV-2 spike protein, 250 ns for the ACE2 dimer, and 150 ns for the ACE2-Estrogen solvated systems (Figure S5a,c,d).

### 4.10. Structure and Data Analysis

All molecular interactions were carried out with a rigid-rigid body docking analysis using PatchDock [58] server to obtain the interacting residues between the S-RBDs and ACE2 proteins. All unacceptable molecular complexes were discarded using the PatchDock algorithm analysis and results were scored by their geometry shape complementarity and effective atomic contact energy [59]. For the molecular docking, the ACE2 protein was indicated as the “receptor” and the spike protein was indicated as the “ligand”. 4.0 Ǻ clustering RMSD and default mode parameters were used to obtain 241 S-RBD-ACE2 structure complexes (Table S6). The steric impediments were calculated based on SARS-CoV-2 spike protein sizes [60,61], which diameter varies from 9 to 12 nm and structures that had steric impediments (intermembranal clashes) were discarded. Statistical results, RMSD, RMSF, RG, SASA, hydrogen bonds, free energies, matches, structures, movies, and b-factor maps were obtained using Gromacs modules. The analysis of the structure’s properties was performed using MD trajectories on the last 50 ns of each simulation, then visualized using Visual Molecular Dynamics (VMD) software [62] and UCSF Chimera v. 1.14 [63]. The graphs were plotted using XMGrace software [64]. FEL maps were used to visualize the energy associated with the protein conformation of the different models during a molecular dynamic simulation. These maps are usually represented by two variables related to the atomic position and one energetic variable, typically the Gibbs free energy. In this work, we considered two substructures of ACE2 protein for FEL map analysis, the Alpha1-2 region (I21 to Y83) and loops regions l2-3 and l3-4 (D303 to R357). The FEL maps were plotted using the gmx sham module while the RMSD and radius of gyration were considered as atomic position variables with respect to its average structure and figures were constructed using Wolfram Mathematica 12.1 [65] (Figure S6a,b).

## Data Availability

Not applicable.

## Supplementary Materials

The following are available online at https://www.mdpi.com/article/10.3390/ijms222111508/s1.
