# Supplementary material for: Structural and Functional Analysis of Female Sex Hormones against SARS-CoV-2 Cell Entry
Source: Int J Mol Sci. 2021 Oct 26;22(21):11508. doi: 10.3390/ijms222111508 (PMC8584232; doi:10.3390/ijms222111508)
Supplement: Supplementary file 1 [file ijms-22-11508-s001.zip › Supplementary Tables.pdf]

Supplementary Tables

Table S1

Spike and ACE2 glycoproteins

Protein	Total residues	Missing residues	N-linked Glycan (Asn)	Interaction sites
**Spike Trimer**	3843[a] (1281[b]) 3363[c] (1121[b]) (Ala27 to Ser1147)[d]	-18 to 26, 70 to 79, 144 to 185, 246 to 262, 445 to 488, 502, 621 to 640, 677 to 688, 828 to 853, 1148 to 1262	61, 74, 122, 149, 165, 234, 282, 331, 343, 603, 616, 657, 709, 717, 801, 1074, 1098, 1134	R-B Domains: T333 - P527
**ACE2 dimer**	1628[a] (814[b]) 1424[c] (712[b]) (Ile21 to Gly732)[d]	-8 to 20, 769 to 805	53, 90, 103, 322, 432, 546, 690	Gln24, Asp30, His34, Tyr41, Gln42, Met82, Tyr83, Gln325, Glu329, Lys353, Arg357

Table S2

Residue Interaction	ACE2 Chain B	Spike Protein Chain B	Spike Protein Chain C
**Hydrogen bonds**	Asp38, Tyr41, Trp48, Gly326		Asn440, Asp442, Ser443, Asn450, Glu484
**Hydrophobic**	Thr27, Lys 31, His34, Glu35, Ala36, Asp38, Leu39, Tyr41, Gln42, Leu45, Trp48, Asn49, Thr52, Asn61, Lys68, Phe72, Asp303, Ala304, Gln305, Thr324, Gln325, Gly326, Phe327, Trp328, Glu329, Asn330, Ser331, Met332, Leu333, Thr334, Asp335, Pro336, Gly337, Lys353, Asp355, Arg357, Ile379	Ile472, Tyr473, Ala475, Gly476, Ser477, Thr478, Pro479, Cys480, Gly482, Val483, Glu484, Asn487	Thr345, Arg346, Asn440, Asp442, Ser443, Lys444, Val445, Gly446, Asn448, Tyr449, Asn450, Tyr451, Pro479, Cys480, Asn481, Val483, Glu484, Gly485, Phe486, Cys488, Tyr489, Phe490

Glycan interactions

	G-N53 (ACE2 glycan)		
	**ACE2 Chain B**	**S-Chain A**	**S-Chain C**
**Glycan - Residues**	Asn49 (HB), Thr55, Glu57 (HB), Asn58, Asn61	Asn456 (HB), Ser457, Asn458 (HB), Leu460, Gln525 (HB)	Val445, Gly446 (HB), Val483 (HB), Gln498, Thr500

	ACE2 glycan	Spike glycan
**Glycan - Glycan**	G-N53, G-N322, G-N546	G-N165, G-N343

Table S3

Stability Descriptors						
**System**	**RMSD**	**[a]RMSD Alignment**	**RMSF**	**Radius of Gyration**	**H Bonds**	
					**Intra**	**[b]Inter**
**Spike**	0.54±0.05	-	0.21±0.12	4.98±0.03	2191.25±29.11	6441.53±81.87
**ACE2-Is**	0.61±0.09	13.73	0.26±0.11	4.03±0.05	1031.44±16.61	2902.94±31.95
**ACE2-E**	0.41±0.09	12.30	0.22±0.08	3.98±0.03	1043.44±16.83	2833.53±41.16
**ACE2-SE**	0.29±0.04	12.81	0.18±0.07	3.99±0.02	1042.02±16.80	2828.15±36.58

Table S4

Interaction	ACE2 protein - 17β-diol	
	Chain A	Chain B
**Polar**	K94, K247, N250, D292, D295, Q305, R306, K309, E310, E312, G422, L424, P688	S257
**Hydrophobic**	N53, Q89, L91, N250, P258, S280, F285, G286, Q 287, K288, N290, D295, A296, D299, Q305, R306, K309, N322, V339, K419, S420, L423, L424, P426, E527, V574, A576, N580, R582, P583, N586, N601, S602, A687, P688	F28, Y41, Q76, T78, Q81, M82, M249, A251, P253, I256, I259, G319, P321, N322, Q325, N330, G354, M383, T548, Q552, F555, P612, Y613, A614

Interaction	ACE2 protein - S-equol	
	Chain A	Chain B
**Polar**	N121, S280, P289, Q380, D431, E527, D609, L664	K31, D67, T92, Q96, N290, G319, D615, R621, K625, R678, E723
**Hydrophobic**	I21, F28, V59, Q60, N63, M82, Y83, K94, L95, T118, T122, Y158, V209, N210, G211, V212, A246, L248, Y252, S254, Y255, N277, L281, Q287, K288, Q305, F308, N322, Q325, W328, M383, L424, D427, F428, T434, G537, L539, S563, P565, N586, E589, P590, T593, Q598, N599, N601, S602, W610, D615, I663, A687, P688, K689, N690, D693	Q24, K26, T27, F28, E35, L39, N64, K68, A71, F72, E75, Q76, T78, L79, Q81, N90, L91, T92, V93, L95, Q98, A99, Q102, L156, W163, S167, R169, S170, H239, I256, S280, G286, Q287, P289, P321, V339, Q388, L392, R393, N432, E435, I436, H540, G551, Q552, F555, R559, S563, E564, P590, T593, K596, N599, K600, N601, S602, V604, P612, Y613, Q616, R621, I622, S623, S626, L628, G629, A632, W635, N636, P677, R678, I679, S680, S721

Table S5

Total possible structures

	ACE2 - Spike docking (75 structures)					ACE2 / Estradiol - Spike docking (95 structures)					ACE2 / S-equol - Spike Docking (71 structures)				
**#**	**No. Sol.**	**Score**	**Area**	**pen.**	**ACE**	**No. Sol.**	**Score**	**Area**	**pen.**	**ACE**	**No. Sol.**	**Score**	**Area**	**pen.**	**ACE**
1	1	14456	4062.7	-4.36	-544.9	1	19344	3276.0	-4.04	-379.2	1	16280	3363.4	-3.99	-623.0
2	2	14364	2317.5	-3.74	150.7	7	15134	2762.1	-4.28	218.7	2	15886	3027.7	-3.88	-443.0
3	5	12958	2958.2	-4.38	-65.8	8	14072	3379.7	-4.27	-651.5	11	11376	2351.0	-3.99	-137.1
4	**9**	**11764**	**2267.3**	**-3.57**	**144.1**	13	11936	1584.0	-3.14	-61.3	12	11326	1380.4	-2.48	-166.0
5	16	10240	1448.7	-3.71	148.9	23	11220	1826.4	-3.78	-365.0	**13**	**11290**	**2896.7**	**-4.59**	**-706.6**
6	26	9348	1340.4	-3.09	8.4	**24**	**11026**	**2006.5**	**-3.53**	**-735.0**	14	11202	2251.6	-3.22	-315.8
7	29	9288	2053.2	-3.81	-213.2	**26**	**10960**	**2743.1**	**-4.07**	**-1119.0**	16	10676	1983.2	-3.62	-215.9
8	36	8764	1223.4	-3.15	49.1	28	10632	2139.6	-3.96	-626.7	**18**	**10554**	**2072.4**	**-3.83**	**-491.0**
9	48	7666	994.4	-2.84	91.3	31	10358	2702.7	-4.38	190.9	20	10226	1681.2	-3.29	158.2
10	50	7382	1728.1	-4.25	-137.0	36	9910	2373.1	-4.1	-349.4	25	9714	1707.3	-4.21	-25.0
11	57	6818	1881.7	-4.27	51.2	47	9218	1608.2	-3.36	-307.7	**27**	**9616**	**1667.9**	**-3.92**	**253.5**
12	58	6756	1215.1	-3.47	32.3	66	7476	997.6	-2.77	-192.2	32	9040	2271.3	-4.59	405.4
13	61	6492	915.6	-3.09	362.0	69	7290	1515.3	-3.74	-194.6	33	9012	2140.6	-4.2	-814.9
14	64	6342	1140.3	-4.07	258.5	70	7240	1013.4	-2.85	-151.8	**39**	**8670**	**1575.4**	**-3.88**	**-176.6**
15	67	6144	1333.2	-3.45	229.9	79	6882	906.3	-2.97	-105.7	43	8382	2328.5	-4.02	-651.7
16	68	6126	1673.1	-3.84	-321.0	81	6786	1748.9	-4.74	-740.1	44	8340	1159.6	-3.46	-264.2
17						82	6744	1881.7	-3.94	-408.8	**47**	**8104**	**1662.4**	**-4.24**	**110.8**
18						89	6392	1423.2	-4.42	37.8	49	7836	2059.5	-4.18	24.2
19						**91**	**6374**	**1211.8**	**-3.94**	**-402.3**	51	7704	1076.6	-2.97	-211.4
20						94	5882	985.4	-3.19	-146.8	54	7258	2019.1	-3.91	-686.1
21											64	6540	1765.9	-4.22	-422.8

Table S6

ACE2 - Spike dock (75 structures)			ACE2 / Estradiol - Spike dock (95 structures)			ACE2 / S-equol - Spike dock (71 structures)		
**No. Sol.**	**Score**	**ACE**	**No. Sol.**	**Score**	**ACE**	**No. Sol.**	**Score**	**ACE**
1	14456	-544.88	1	19344	-379.16	1	16280	-622.98
2	14364	150.68	2	17450	-631.80	2	15886	-443.04
3	14118	202.45	3	17064	46.43	3	14982	186.01
4	13380	-699.27	4	15692	409.33	4	13398	-448.47
5	12958	-65.79	5	15370	-327.04	5	13292	186.93
6	12916	118.07	6	15146	-816.85	6	13152	96.99
7	11906	306.25	7	15134	218.73	7	12978	-188.93
8	11820	142.59	8	14072	-651.50	8	12536	386.91
9	11764	144.13	9	13528	-260.32	9	11668	125.24
10	11214	0.33	10	12892	-127.96	10	11532	-131.08
11	11068	237.85	11	12132	93.40	11	11376	-137.07
12	10806	-151.31	12	12108	-160.08	12	11326	-165.97
13	10694	185.03	13	11936	-61.34	13	11290	-706.61
14	10440	230.18	14	11894	-289.35	14	11202	-315.79
15	10254	277.32	15	11582	-288.00	15	11152	-868.97
16	10240	148.93	16	11578	42.08	16	10676	-215.87
17	10098	66.53	17	11542	-153.44	17	10610	127.39
18	10032	-665.78	18	11536	139.82	18	10554	-490.98
19	9882	252.60	19	11380	250.84	19	10258	137.11
20	9840	-168.06	20	11318	-652.64	20	10226	158.24
21	9716	260.61	21	11278	64.42	21	10052	321.09
22	9578	-650.44	22	11224	-446.55	22	9924	-19.79
23	9482	96.71	23	11220	-365.04	23	9890	134.41
24	9440	-221.94	24	11026	-735.03	24	9786	-4.51
25	9366	164.42	25	11014	-537.85	25	9714	-24.96
26	9348	8.44	26	10960	-1119.00	26	9700	-265.77
27	9322	271.79	27	10916	1.34	27	9616	253.46
28	9298	55.22	28	10632	-626.67	28	9434	307.00
29	9288	-213.17	29	10612	129.21	29	9280	-624.63
30	9282	119.62	30	10372	-724.63	30	9236	-5.88
31	9264	-544.50	31	10358	190.86	31	9054	-765.69
32	9084	-143.53	32	10314	-727.60	32	9040	405.39
33	9044	143.66	33	10284	-463.39	33	9012	-814.93
34	9000	185.97	34	10192	-204.63	34	8906	40.69
35	8914	-114.87	35	9992	-438.68	35	8894	-627.36
36	8764	49.13	36	9910	-349.35	36	8850	221.04
37	8724	456.41	37	9804	-105.85	37	8838	-67.58
38	8572	412.82	38	9802	83.42	38	8716	-7.88
39	8568	-572.24	39	9776	-326.99	39	8670	-176.62
40	8296	-24.52	40	9652	-340.99	40	8652	-541.82

Continuation

ACE2 - Spike dock (75 structures)			ACE2 / Estradiol - Spike dock (95 structures)			ACE2 / S-equol - Spike dock (71 structures)		
No. Sol.	Score	ACE	No. Sol.	Score	ACE	No. Sol.	Score	ACE
41	8296	-576.53	41	9616	260.50	41	8576	-103.06
42	8286	70.92	42	9450	-174.07	42	8426	-143.84
43	8238	-9.11	43	9416	-91.08	43	8382	-651.69
44	8198	-117.47	44	9362	71.03	44	8340	-264.22
45	7984	28.89	45	9360	-261.54	45	8214	-39.21
46	7824	190.58	46	9340	-107.16	46	8116	266.73
47	7730	-15.20	47	9218	-307.71	47	8104	110.79
48	7666	91.33	48	9212	-124.38	48	7874	-691.07
49	7448	83.66	49	9184	342.39	49	7836	24.22
50	7382	-136.97	50	8904	-392.49	50	7722	49.59
51	7282	107.13	51	8860	-139.93	51	7704	-211.40
52	7244	-110.98	52	8852	-332.36	52	7522	262.71
53	7192	167.97	53	8722	-696.22	53	7396	188.03
54	7174	139.88	54	8508	-168.91	54	7258	-686.06
55	6892	69.24	55	8462	-99.89	55	7158	-157.70
56	6866	-214.11	56	8456	-312.59	56	6992	97.24
57	6818	51.19	57	8448	-247.11	57	6880	314.22
58	6756	32.29	58	8316	-71.91	58	6866	-252.80
59	6654	-69.60	59	8076	-951.26	59	6782	194.00
60	6514	-25.93	60	8054	-269.20	60	6774	-134.68
61	6492	361.97	61	8002	-54.94	61	6744	350.17
62	6472	236.13	62	7924	-49.78	62	6612	168.70
63	6428	-54.25	63	7820	278.16	63	6580	-198.15
64	6342	258.48	64	7648	-201.18	64	6540	-422.75
65	6306	268.38	65	7506	238.83	65	6478	381.46
66	6160	330.79	66	7476	-192.24	66	6226	107.35
67	6144	229.93	67	7344	-198.69	67	5990	33.03
68	6126	-320.99	68	7306	-85.64	68	5960	-344.88
69	6036	-131.23	69	7290	-194.60	69	5938	238.01
70	5856	-26.07	70	7240	-151.81	70	5888	-518.96
71	5388	-125.73	71	7218	-440.81	71	5044	-83.43
72	5362	-313.25	72	7192	-49.49			
73	5284	169.23	73	7162	-185.84			
74	5200	345.23	74	7144	-253.15			
75	4922	10.63	75	7112	-292.61			
			76	6944	139.06			
			77	6938	-105.11			
			78	6906	-182.34			
			79	6882	-105.73			
			80	6872	-3.24			

Continuation

ACE2 - Spike dock (75 structures)			ACE2 / Estradiol - Spike dock (95 structures)			ACE2 / S-equol - Spike dock (71 structures)		
No. Sol.	Score	ACE	No. Sol.	Score	ACE	No. Sol.	Score	ACE
			81	6786	-740.06			
			82	6744	-408.83			
			83	6666	337.03			
			84	6664	246.32			
			85	6544	-285.73			
			86	6440	-56.10			
			87	6436	116.17			
			88	6432	-129.70			
			89	6392	37.80			
			90	6384	114.78			
			91	6374	-402.26			
			92	6174	10.90			
			93	6110	-177.81			
			94	5882	-146.83			
			95	5844	-27.46			
